# ATLL introduction: is there a standard of care for treatment of adult T cell Leukemia Lymphoma?

**DOI:** 10.1186/1742-4690-11-S1-O30

**Published:** 2014-01-07

**Authors:** Ali Bazarbachi, Felipe Suarez, Ambroise Marcais, Olivier Hermine

**Affiliations:** 1 Department of Internal Medicine, American University of Beirut, Beirut, Lebanon; 2 CNRS UMR 8147 and Department of Hematology, Necker Hospital, Paris Descartes University, Paris, France

## 

ATL carries a very bad prognosis because of intrinsic chemoresistance and severe immuno-suppression. In acute ATL, chemotherapy combinations improved response rate but failed to achieve a significant impact on survival. Patients with chronic and smoldering ATL have a better prognosis but long-term survival is poor when patients are managed with a watchful-waiting policy or with chemotherapy. The antiviral combination of zidovudine (AZT) and interferon-alpha (IFN) improves survival in leukemic subtypes of ATL (smoldering and chronic ATL and acute ATL with wild type p53) and should be considered as standard first line therapy. However, it is mandatory to 1) use it in leukemic forms as first line therapy and not after one or more cycles of chemotherapy; 2) start with high doses of both agents since reduced doses are often not effective. ATL lymphoma may benefit from initial chemotherapy combined to or followed by AZT/IFN and this approach should be tested in prospective clinical trials. Prophylaxis of opportunistic infections and intrathecal chemotherapy are mandatory. Yet, most patients relapse and alternative therapies are mandatory. IFN and arsenic trioxide induce Tax proteolysis, synergize to induce apoptosis in ATL cells and cure Tax-driven ATL in mice through specific targeting of leukemia initiating cell activity. Therefore, to prevent relapse, clinical trials assessing consolidative targeted therapies such as arsenic/IFN combination, histone deacetylase inhibitors or novel monoclonal antibodies particularly brentuximab in CD30 positive cases or the promising anti CCR4 antibodies, are mandatory after achieving CR with AZT/IFN. Finally, allogeneic BMT should be considered in suitable patients.

